# *In vitro* evaluation of anti-infective activity of a *Lactobacillus plantarum* strain against *Salmonella enterica* serovar Enteritidis

**DOI:** 10.1186/1757-4749-5-11

**Published:** 2013-05-13

**Authors:** Jugal Kishore Das, Debasmita Mishra, Pratikshya Ray, Prangya Tripathy, Tushar K Beuria, Neera Singh, Mrutyunjay Suar

**Affiliations:** 1 School of Biotechnology, KIIT University, Bhubaneswar 751024, India; 2 Institute of Life Sciences (ILS), Bhubaneswar 751024, India

**Keywords:** Probiotic, *Lactobacillus*, *Salmonella*, HCT-116, Cell free culture supernatant

## Abstract

**Background:**

*Salmonella enterica* serovar Enteritidis infections are known to exhibit worldwide prevalence with increased morbidity and mortality. The conventional strategies like antibiotic therapy and vaccination have not only proved to be of sub-optimal efficacy but also led to the development of multidrug resistant strains of *Salmonella*. Antimicrobial activities of probiotics against various enteropathogens and other health promoting effects have assumed greater significance in recent years. The present study aims to evaluate the efficacy of a *Lactobacillus plantarum* strain (KSBT 56, isolated from a traditional food product of India), in preventing *Salmonella enterica* serovar Enteritidis growth and pathogenicity *in vitro*.

**Methods and results:**

The cell free culture supernatant (CFCS) of KSBT 56 strain notably inhibited the growth of *Salmonella* Enteritidis without affecting the growth of other gram-positive lactic acid bacteria. The isolated KSBT 56 strain produces lactic acid similar to other standard probiotic strains like *Lactobacillus plantarum* MTCC 1407. The free radical production by KSBT 56 strain was studied by using *sodC* mutant of *S.* Enteritidis, which exhibited reduced growth in the presence of CFCS of the KSBT 56 strain, indicating the inhibitory activity of free radicals on the growth of *S*. Enteritidis. Our results also showed a significant reduction in the biofilm forming ability of *Salmonella* Enteritidis in the presence of the KSBT 56 strain (2 log cfu/ml, p = 0.01). Further, the anti-infective characteristics of KSBT 56 strain was validated by gentamicin protection assay which revealed 80% reduction in the invasion of *Salmonella* Enteritidis to HCT-116 cell line (*Salmonella* Enteritidis and KSBT 56 in a 1:1 ratio) and delayed addition of *Salmonella* Enteritidis by 1 h. Similarly, the reduced adhesion of *Salmonella* to the HCT-116 cells was observed along with the down regulation of *hilA* gene of *Salmonella* Pathogenicity Island 1 (SPI1) indicating that they might have acted synergistically to decrease the invasion of the pathogen into the cell line.

**Conclusions:**

KSBT 56 strain effectively inhibited the growth, invasion and the biofilm forming ability of *Salmonella* Enteritidis without inhibiting the growth of other *Lactobacillus* strains. Overall, our result suggested that KSBT 56 can be used as a potential probiotic strain with considerable beneficial effects on the host.

## Background

*Salmonella* enterica is a major food borne pathogen and one of the leading causes of serious illness ranging from acute gastroenteritis to systemic infections like typhoid. Infections with non typhoidal serovars of *Salmonella* enterica, predominantly *Salmonella* Enteritidis (*S.* Enteritidis) and *S*. Typhimurium are more frequent and occur in both developing and industrialized nations. These infections are primarily associated with gastrointestinal inflammation and diarrhea and are generally self-limiting [1]. The established strategies to combat *Salmonella* infections include vaccination and the use of antibiotics. However, the frequent and prolonged use of antibiotics not only leads to increasing antibiotic resistance among *Salmonella* serovars but also alters the intestinal commensal flora [2]. The emergence of multidrug resistant strains and the suboptimal efficacy of currently available vaccines have necessitated the search for alternative therapies against *Salmonella* infections [3-5]. One such promising alternative is the possible therapeutic use of probiotics against various enteropathogens [6-8].

Probiotics are defined as “live microorganisms which when administered in adequate amounts confer a health benefit on the host” [3]. The possible mechanisms by which probiotics may inhibit enteric pathogens include modification of the host intestinal environment and immune system, competition for nutritional substrates as well as sites of adhesion on intestinal epithelial cells, secretion of antimicrobial compounds and inactivation of toxins [4]. Earlier studies have reported the use of probiotics in the prevention and treatment of gastrointestinal infections caused by *Salmonella*[5]. However, the underlying molecular mechanisms by which probiotics offer protection against gastrointestinal pathogens are not fully elucidated [9-11]**.** The most extensively studied probiotic strains are reported from genera *Lactobacillus* and *Bifidobacterium*, which are also included in many functional foods and dietary supplements [12,13]. The beneficial effects of the probiotics are known to be genus, species and strain specific and a particular probiotic strain is found to be active against selected enteric pathogen only [9,14,15]. The selection of a microbial strain is therefore, an important criterion to consider it as a probiotic for its effective and potential therapeutic use.

Food based probiotics have assumed greater significance in recent years as different food products can harbor native and beneficial *Lactobacilli* and thus can be used for both nutritional and therapeutic purposes. Traditional Indian foods are well known for their unique fermentation style and can be used as a source of potentially beneficial probiotics. The antibacterial mechanisms of action of these *Lactobacillus* strains, especially the production of nonbacteriocin molecules, have not been extensively studied. The main objective of the present study is to determine the efficacy of an isolated probiotic strain in preventing *S.* Enteritidis infections. Further, the mechanism of antimicrobial activity was assessed in order to establish it as a potential probiotic strain, specifically active against *S.* Enteritidis, which contributes to major *Salmonella* infections.

## Results

### Effect of CFCS on viability of *S. Enteritidis*

**Figure 1:**
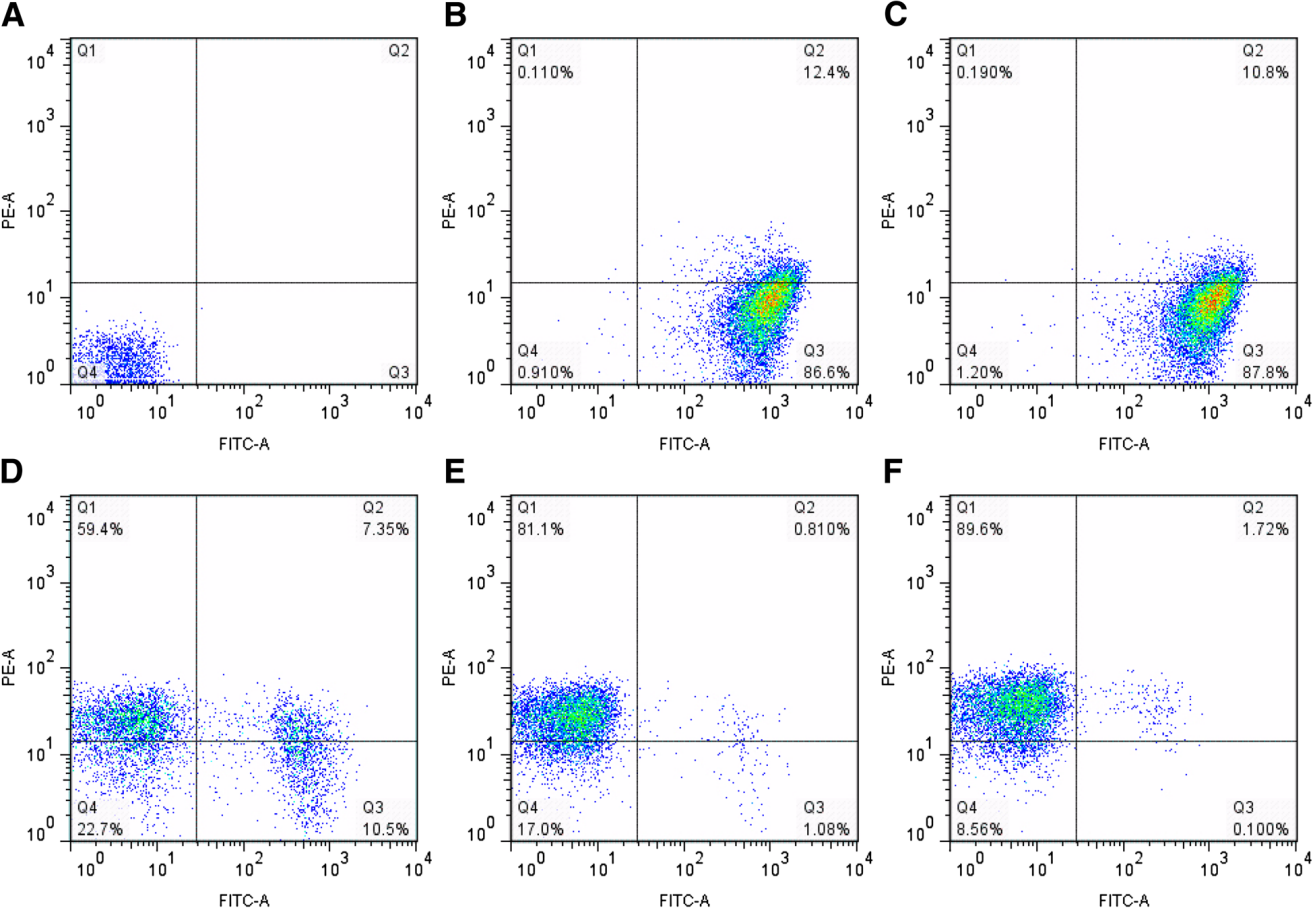
**Flow cytometer analysis of live/dead *S. *Enteritidis grown in the CFCS of KSBT 56 strain. ***S.* Enteritidis expressing GFP are shown in Q3 in FITC channel. Propidium Iodide positive *S.* Enteritidis is shown in Q1 in PE-A channel. *S.* Enteritidis with compromised membrane expressing both GFP and propidium Iodide are seen in Q2. **A**. Untreated *S.* Enteritidis is shown in Q4. **B.***S.* Enteritidis treated with 3% CFCS shows 86.6% of the population expressing GFP. **C.***S.* Enteritidis treated with 5% CFCS has 87.8% of the population expressing GFP. **D.***S.* Enteritidis treated with 7% CFCS shows 10.5% live S. Enteritidis expressing GFP. **E.***S*. Enteritidis treated with 9% CFCS shows 1.08% live *Salmonella* in Q3 **F.***S.* Enteritidis treated with 11% CFCS shows 0.1% live *S.* Enteritidis in the GFP-positive quadrant (Q3).

The CFCS of *Lactobacillus* strains are known to have antimicrobial effects against enteric pathogens [14]. In this study, the effect of CFCS of KSBT 56 strain on *S.* Enteritidis viability was assessed using flow cytometric analysis. The dead bacterial cells were shown in the propidium iodide quadrant (Figure 1). Different concentrations (3%, 5%, 7%, 9% and 11%) of CFCS of KSBT 56 were used to study its effect on the viability of *S.* Enteritidis. The inhibition of *S.* Enteritidis increased with increasing concentration of the CFCS of the probiotic strain. Effective killing of *S.* Enteritidis (89.6%) was observed with 11% of CFCS after 4 h. Similarly, CFCS was also found to be effective against other pathogens such as *E. coli*, *S*. Typhi and *S*. Typhimurium (data not shown).

### Effect of CFCS of the KSBT 56 on other *Lactobacillus strains*

**Figure 2:**
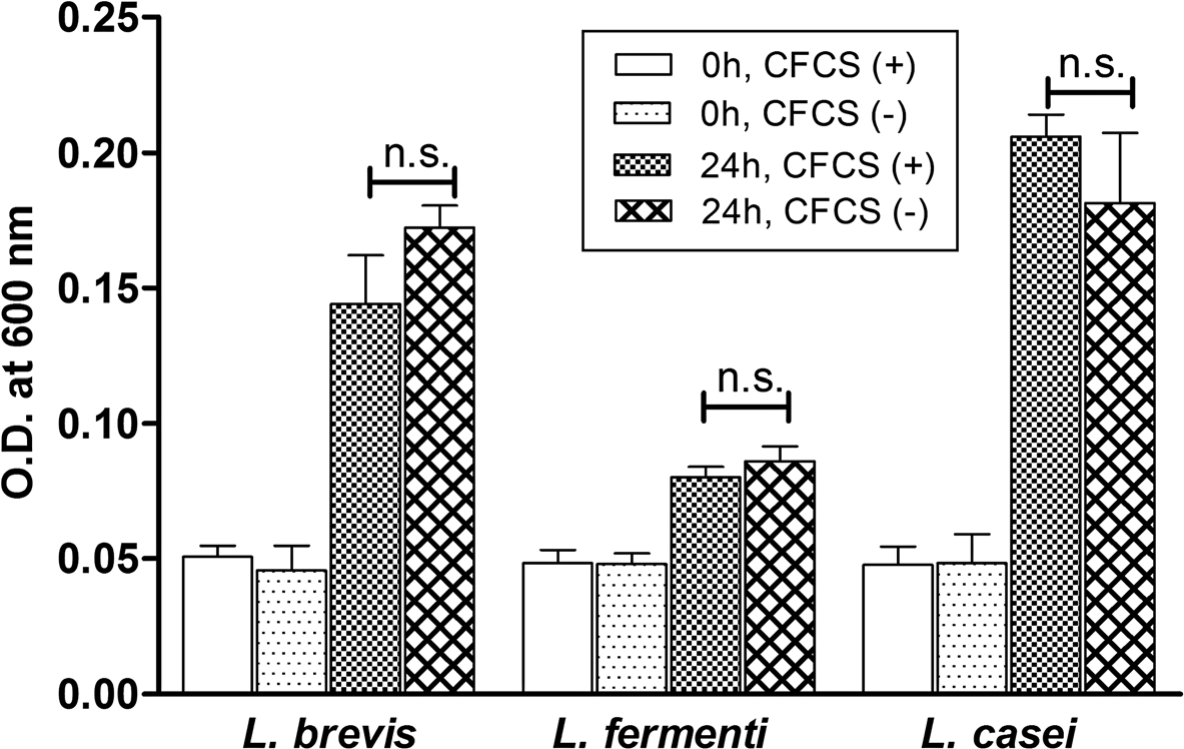
**The growth of different *Lactobacillus *strain in the presence of CFCS of KSBT 56 strain.** The absorbance of the cultures at 600 nm is plotted on the y-axis. The growth of different *Lactobacillus* strains was analysed by comparing absorbance at 0 h and 24 h of growth in presence of CFCS of KSBT 56.

Probiotics should be able to selectively inhibit pathogens while not having any deleterious effect on the normal gut flora, to be considered as safe for consumption. The effect of CFCS of the KSBT 56 strain was studied by incubating different *Lactobacillus* strains with the probiotic CFCS. No significant differences were observed in the viability of the standard *Lactobacillus* strains in the presence or absence of the CFCS (Figure 2). The results partly confirm the safety of KSBT 56 as a probiotic strain as it does not have a deleterious effect on the normal commensal gut flora.

### Production of lactic acid by KSBT 56

Lactic acid is one of the important factors produced by *Lactobacillus* strains which inhibit various pathogens at a specific concentration. For example, the standard *Lactobacillus* strain *Lactobacillus plantarum* (*L. plantarum*) MTCC 1407 inhibits pathogens like *Salmonella* and *Shigella*, at a concentration of 6.0 mM lactic acid produced at 6 h. In the present study, the lactic acid concentration of KSBT 56 was estimated to be 5 mM at 6 h, which was comparable to that of *L. plantarum* MTCC 1407. The total lactic acid concentration in the CFCS of KSBT 56 was also estimated to be 5mM at 6h, indicating its antimicrobial property.

### Inhibitory effect of free radicals produced by KSBT 56 on *S. Enteritidis*

**Figure 3:**
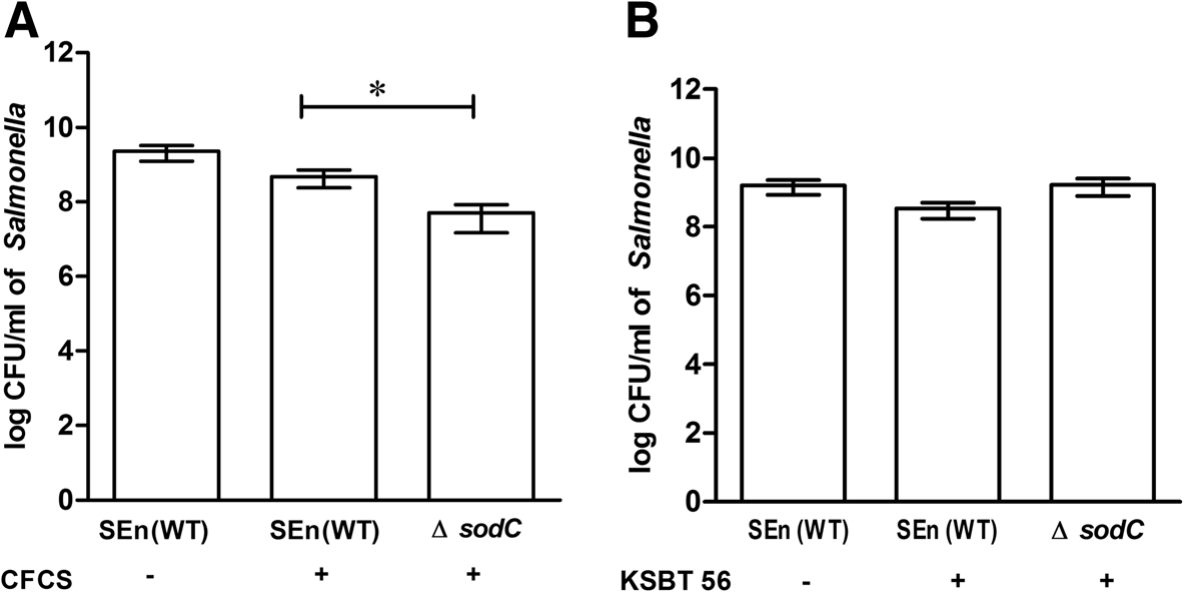
**Inhibition of growth of *S. *Enteritidis WT and Δ*sodC *mutant in the presence of CFCS (A) of KSBT 56 or live KSBT 56 (B). A.***S.* Enteritidis (SEn) WT or a mutant strain deficient of *sodC* gene (*ΔsodC*) were co-incubated with CFCS. **B**. The above groups were also co-incubated with live KSBT 56 bacterial culture. The cfu was enumerated by plating on LB agar plates supplemented with streptomycin. The presence of CFCS or KSBT 56 is indicated by (+) and the absence is indicated by (−). The growth of Δ*sodC* is compared with *S*. Enteritidis WT strain grown in the presence of CFCS or live KSBT 56 strain.

The antimicrobial activity of the free radicals produced by KSBT 56 strain against *S.* Enteritidis was determined by using superoxide dismutase (*sodC*) gene knock out mutant*.* A *sodC* mutant is known for its increased susceptibility to free radicals as compared to the wild type (WT) strain. Results showed that *S.* Enteritidis harboring *sodC* mutation exhibited reduced growth in the presence of CFCS of the KSBT 56 strain in the co-culture experiment (Figure 3). As compared to the *S*. Enteritidis (WT) strain, Δ*sodC* mutant was sensitive to CFCS treatment showing a 2-log decrease in its viability on the addition of CFCS (p = 0.01). This indicates that *S*. Enteritidis is susceptible to the free radicals produced by the KSBT 56 strain.

### Inhibitory effect of KSBT 56 on biofilm formation ability of *S. Enteritidis*

**Figure 4:**
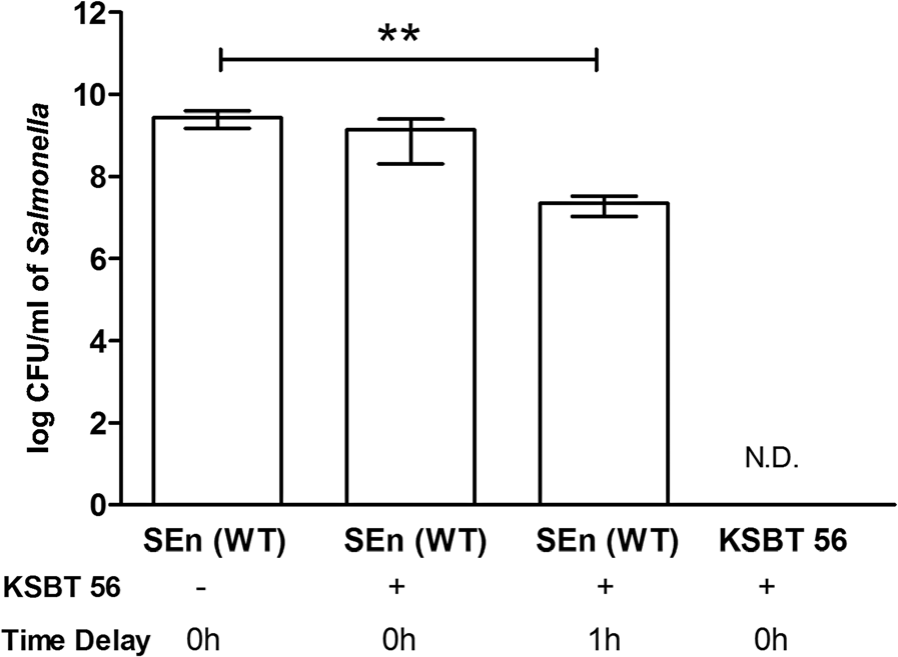
**Inhibition of biofilm formation of *S. *Enteritidis by the KSBT 56 strain.** The biofilm forming colonies of *S.* Enteritidis were enumerated on streptomycin LB Agar plates. The KSBT 56 bacterial culture was added to *S.* Enteritidis either simultaneously (0 h) indicated by (+) or at a time delay of 1 h. The absence of KSBT 56 is denoted by (−). KSBT 56 bacterial culture is plated on streptomycin LB Agar plates as control.

The effect of KSBT 56 on the biofilm forming ability of *S.* Enteritidis was determined by co-culture experiment and by delayed addition of *Salmonella* to KSBT 56 strain in a 96 well plate. Biofilm formation was confirmed by crystal violet staining (data not shown). The cfu recovered from the biofilm formed by *Salmonella* in a 96 well plate were plated on LB agar plates in different dilutions. The simultaneous addition of *S.* Enteritidis with the KSBT 56 strain did not show any significant inhibition of the biofilm formation by *S*. Enteritidis. However, on the delayed addition (1 h) of *S.* Enteritidis to the culture containing the probiotic strain, a 2-log decrease in biofilm forming colonies of *Salmonella* was observed (p = 0.01) (Figure 4).

### Inhibition of invasion of *S. Enteritidis* by KSBT 56

**Figure 5:**
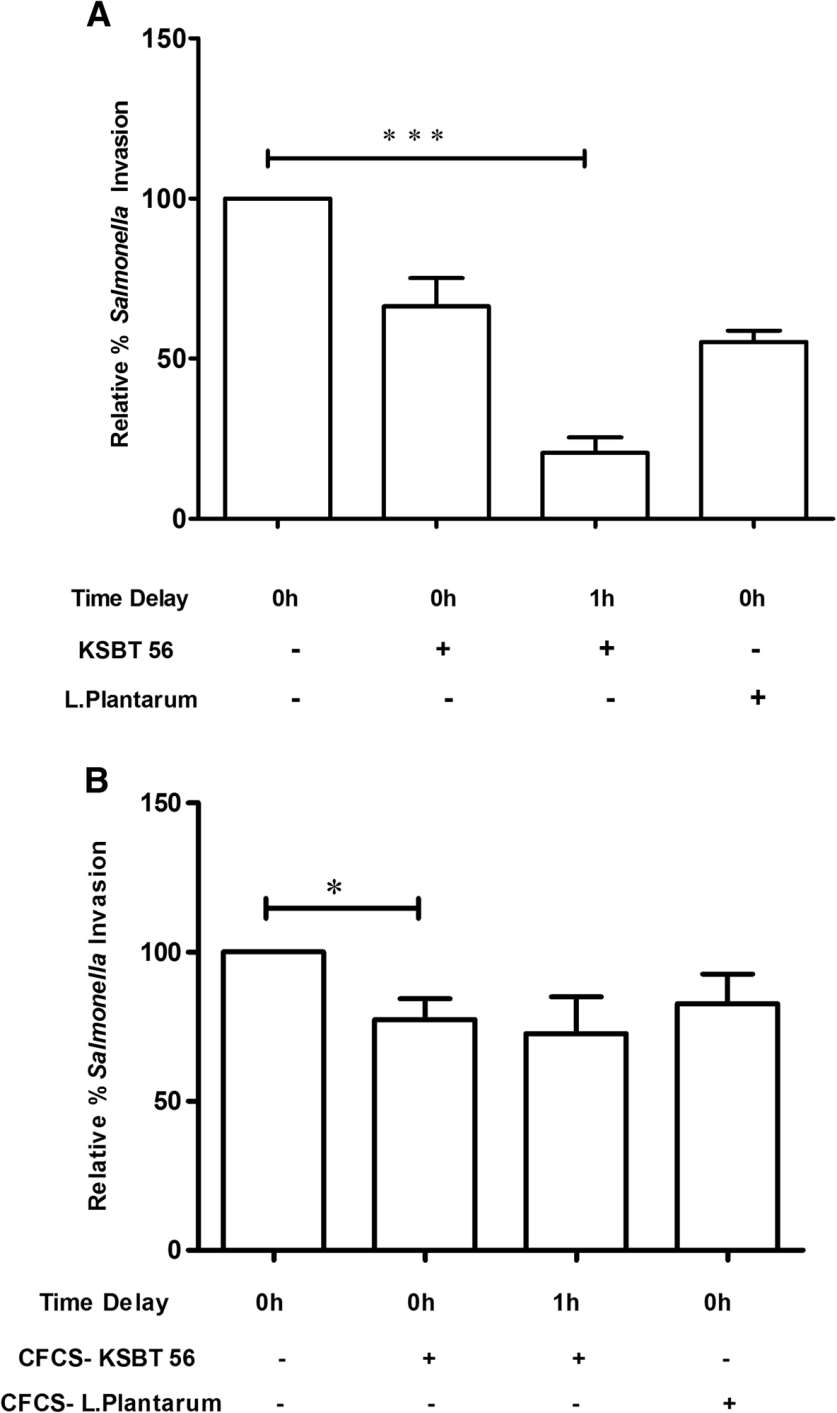
**Effect of KSBT 56 on invasion of *S. *Enteritidis (A) and effect of CFCS of KSBT 56 on invasion of *S. *Enteritidis to HCT-116 cells. A.** Gentamicin protection assay was performed to determine the invasion of *S*. Enteritidis into the HCT-116 cell line in the presence (+) or absence (−) of KSBT 56 strain. The pathogen and the KSBT 56 strain were either co-infected together into the cell line (0 h) or the pathogen was added at a time delay of (1 h). **B.** The effect of CFCS on invasion of *S*. Enteritidis was determined by co-incubating *S*. Enteritidis with the CFCS of KSBT 56 in 24- well tissue culture plate seeded with HCT-116 cell line. *S*. Enteritidis was also cultured with CFCS for 1 h before infection of HCT-116 cells. *L. plantarum* MTCC 1407 was taken as a reference strain. The invasion of *S*. Enteritidis to HCT-116 cells is taken as control.

**Figure 6:**
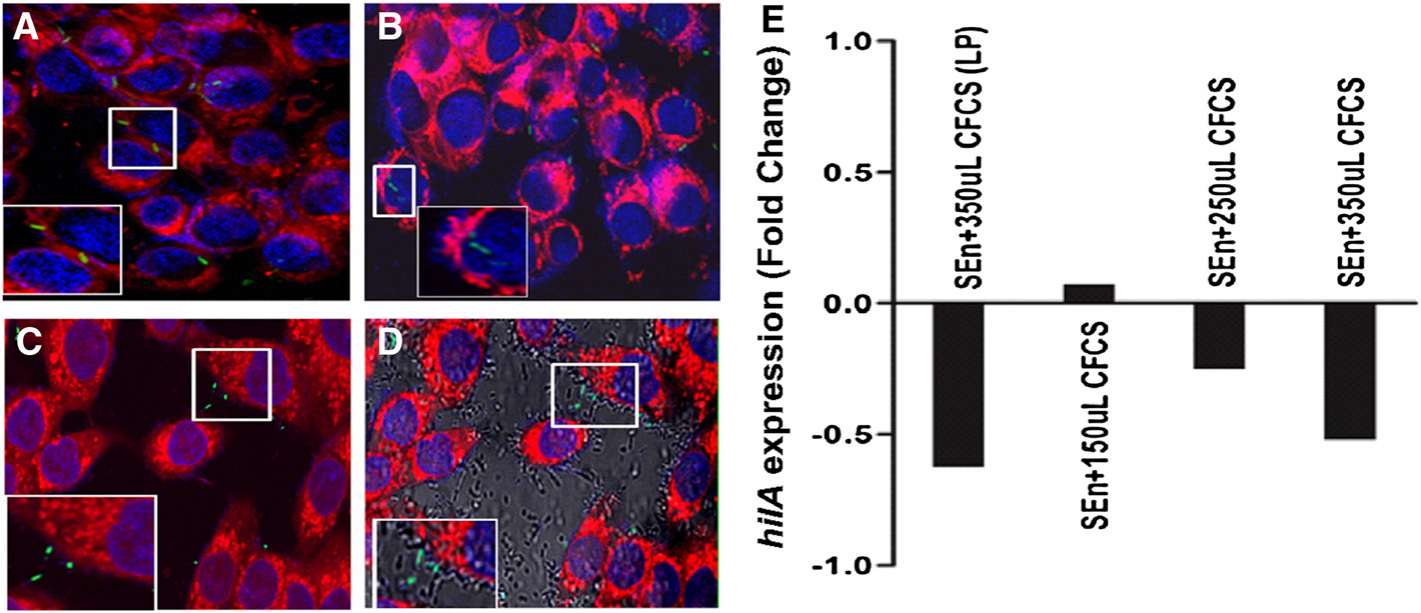
**Confocal images of *Salmonella *invasion (A-D) and Expression of *hilA *gene by RT-PCR (E).** Confocal images were taken at 63X magnification using Leica CLSM. The membrane of HCT-116 cell lines were stained with plasma red dye and *S*. Enteritidis was tagged with GFP. The KSBT 56 strain was observed in phase contrast. **A.***S*. Enteritidis invasion into HCT-116 in the absence of KSBT 56 strain. **B.***S*. Enteritidis coinfected with KSBT 56 strain into HCT-116 cell line shows reduced invasion of *S.* Enteritidis. **C.** Delayed addition of *S.* Enteritidis after addition of KSBT 56 strain by 1 h further reduces the invasion of *Salmonella* into the cell lines. **D.** Merged image of panel C with phase contrast shows KSBT 56 adhering to HCT-116. **E.** RT-PCR analysis of *hilA* gene of *S*. Enteritidis grown in increasing concentration of CFCS of KSBT 56 strain. *L. plantarum* MTCC 1407 is a reference strain. The fold change in the expression of *hilA* gene is compared to *S.* Enteritidis WT (Untreated). SEn refers to *S.* Enteritidis and LP refers to *L. plantarum* MTCC 1407.

To determine the inhibitory effect of KSBT 56 on invasion of *S*. Enteritidis, standard gentamicin protection assay was performed with simultaneous and delayed addition of *S*. Enteritidis strain to HCT-116 cell line. Gentamicin kills the extracellular bacteria while the intracellular bacteria are plated on LB agar plates and cfu enumerated. Reduced invasion (by 40%) of *S.* Enteritidis was observed on simultaneous addition of the pathogen and the probiotic strain in the ratio of 1:1 (Figure 5A). Further, the invasion efficiency of *S.* Enteritidis was significantly reduced by 80% on addition of KSBT 56 strain 1 h prior to the addition of *S.* Enteritidis as compared to the control (*S*. Enteritidis only) (p = 0.0012). Similarly, the invasion of *Salmonella* was reduced by 23% on co-incubation with CFCS of KSBT 56 strain and by 28% on delayed addition of *S.* Enteritidis after incubation of the pathogen with CFCS for 1 h (Figure 5B). The confocal images provide further conclusive evidence of the reduced invasion of *S.* Enteritidis and adherence of the KSBT 56 strain to the HCT-116 cell line (Figure 6A-D).

### Adhesion of *S. Enteritidis* to HCT-116 cell line in the presence or absence of KSBT 56

Probiotics are known to adhere to intestinal epithelial cells thereby competitively excluding the adhesion of pathogens. The adhesion of *S*. Enteritidis to HCT-116 colon epithelial cell line was studied by simultaneous and delayed addition of *S*. Enteritidis and KSBT 56 strain. No significant reduction in the adhesion of the *S*. Enteritidis to HCT-116 cells was observed on simultaneous addition of the probiotic and the pathogenic strain. However, the adhesion of *S.* Enteritidis to HCT-116 cell line was significantly reduced (p=0.01) on the delayed infection of *Salmonella* by 1 h after the addition of the KSBT 56 strain. The CFCS of KSBT 56 strain did not decrease the adhesion of *S.* Enteritidis to HCT-116 cell line significantly either on co-incubation or on delayed addition of the pathogen, after 1 h of incubation with CFCS of KSBT 56 strain. The percentage of adhesion of KSBT 56 and *S*. Enteritidis is shown in Table 1.

**Table 1:** **Adhesion of *S. *Enteritidis and KSBT 56 to HCT-116 cell line**

**Infection of HCT-116 cell line**	**Adhesion percentage of SEn**	**Adhesion percentage of KSBT 56**
*S.* Enteritidis WT : KSBT 56 (1:1)	11.68% ± 3.68	6.43% ± 1.69
*S.* Enteritidis WT : KSBT 56 (1:1) delayed addition of *S.* Enteritidis by 1 h	7.7% ± 1.09^a^	7.53% ± 2.4
*S.* Enteritidis WT	12.08% ± 1.51^b^	N.T
KSBT 56	N.T	8.0% ± 2.34
*S.* Enteritidis + CFCS of KSBT 56	10.35% ± 3.50	N.T
*S.* Enteritidis incubated with CFCS of KSBT 56 for 1h	10.06 ±1.06	N. T

The values represent the S.D of 3 different experiments. NT is not tested. SEn is *Salmonella* Enteritidis.

The significant differences between a and b were calculated using *t*-test.

### Effect of CFCS on hilA (SPI1) expression

SPI1 encodes genes which are involved in the invasion of *Salmonella* into intestinal epithelial cells**.** The *hilA* gene, a major transcriptional regulator of SPI1, is reportedly down regulated in the presence of probiotic CFCS [16]. RT-PCR was used to study *hilA* gene expression in the presence of varying concentrations of CFCS of KSBT 56 strain. The results showed that with the increasing concentration of CFCS of the KSBT 56 strain, *hilA* gene expression was consistently down regulated. The *hilA* gene expression in the presence of CFCS of KSBT 56 strain is shown in Figure 6E. Thus, apart from reduced adhesion, the down regulation of *hilA* gene was also responsible for the reduced invasion of *S.* Enteritidis to HCT-116 cells as shown in Figure 6A-D.

## Discussion

Probiotics have been successfully used for the prevention and treatment of various gastrointestinal diseases of human and animals [17]. The beneficial *Lactobacillus* strains present in the fermented dairy products are known to have a nutritional and therapeutic effect on human health [18]*.* Several *in vivo* and *in vitro* studies have demonstrated that probiotics can inhibit *Shigella dysenteriae*[19], *Salmonella*[5] and *Clostridium difficile*[20] associated diarrhea. However, the basis of their mode of action has largely remained unanswered. The present study, therefore aimed to understand the underlying mechanism of action of a novel *Lactobacillus* strain isolated from a fermented milk product.

In this study, the CFCS of the isolated KSBT 56 strain, inhibited *S.* Enteritidis growth in *in vitro* culture system and the live KSBT 56 culture effectively prevented its attachment and invasion to the colon epithelial cell lines (HCT-116). Flow-cytometric dead/live staining analysis is a sensitive measure of bacterial cell death. Therefore the technique has been used in our study to assess *S*. Enteritidis viability when cultured in the presence of CFCS of KSBT 56 strain. By increasing the concentration of CFCS, viable counts of *S*. Enteritidis decreased consistently, but at low concentration, a significant percentage of *S*. Enteritidis were also observed showing positive for both GFP and propidium iodide. This is probably because of their compromised status of the membrane integrity. Similarly, no deleterious effects were observed on the other commensal gut flora further establishing its safety profile. Previous studies have reported that CFCS of *L. plantarum* induces complete inhibition of *Salmonella* growth, which was mainly attributed to the lactic acid production by the probiotic strain [11,21]. CFCS of probiotic *Lactobacillus* strains reportedly contain several antimicrobial compounds [22], lactic and non-lactic acids as well as hydrogen peroxide which can kill various enteropathogens [23]. Earlier studies have also established that lactic acid production by probiotic *Lactobacillus* strains is a key mechanism involved in inhibiting bacterial growth [24,25]. In the present study, the lactic acid produced by the KSBT 56 strain was comparable to that of the reference strain *L. plantarum* and therefore we suggest that the inhibitory activity shown against *S.* Enteritidis could be partly due to the production of lactic acid in the CFCS. Alternatively, the mechanism of the antimicrobial activity of probiotic *Lactobacillus* strains might also include production of other non-lactic acid components and peroxide radicals [17,25]. Furthermore, to study the effect of free radical produced by the strain KSBT 56, *sodC* mutation was incorporated in *S.* Enteritidis. Results showed the increased inhibition of growth of Δ*sodC* mutant when cultured in the presence of CFCS, indicating that free radicals might be generated by the KSBT 56 strain.

One of the reasons *Lactobacilli* have been widely studied is because of their remarkable ability to inhibit the growth of various pathogens by producing antimicrobial compounds and inhibiting biofilm formation by various pathogens [26]. The inhibitory effect of *Lactobacillus* supernatant on biofilm formation by *K. pneumonia* was shown in a recent study [27]*.* Similarly, in another related study, the inhibitory effect of *L. fermentum* supernatant was observed on the *Klebsiella* growth and biofilm formation [28]. In the present study, similar results were observed, where the biofilm forming ability of the pathogen was reduced on delayed addition of *Salmonella,* in the presence of KSBT 56 strain.

One of the key steps identified in the pathogenesis of intestinal pathogens is their ability to attach to the surfaces of intestinal epithelial cells via fimbriae or pili, present on the bacterial cell surface [29]. The subsequent step in *Salmonella* pathogenesis after attachment is the invasion of intestinal epithelial cells. In the present work, colon epithelial cell line, HCT-116 was used to study the adherence and invasion of *S*. Enteritidis*.* Our results demonstrated a significant reduction in the adherence of *Salmonella* to the HCT-116 cell lines when incubated with the KSBT 56 strain. Similarly, invasion of *S*. Enteritidis to HCT-116 cell line was noticeably reduced both in the co-culture experiment and on the delayed addition of *Salmonella*. The significant reduction in invasion of *S*. Enteritidis on delayed addition of the pathogen may be due to the initial attachment of KSBT 56 strain to colon epithelial cells further preventing the attachment of *Salmonella*. Previous studies have also reported reduced adhesion of pathogens in the presence of probiotic strains due to competitive exclusion of the pathogens [30]. However, in the present study we observed reduced invasion of *Salmonella* to HCT-116 cells by 40% in co-culture experiment, although there was no significant difference in the adhesion of KSBT 56. The CFCS of KSBT 56 strain also inhibited the invasion of *Salmonella*, while not having any significant effect on the adhesion of the pathogen to HCT-116 cell line. These results collectively indicate the involvement of an alternative mechanism besides the competitive exclusion of the pathogen, thereby reducing the invasion of *S*. Enteritidis into colon epithelial cell lines.

The invasion of intestinal epithelial cells by *Salmonella* requires a set of genes present on the SPI1, the expression of which is tightly regulated by *hilA*[31]. Previous studies have reported that various probiotic components down regulate *hilA* gene expression in *S.* Typhimurium thereby preventing its invasion into intestinal epithelial cells [16,31-33]. Our data was also in agreement with these findings and showed down regulation of *hilA* gene expression of *S.* Enteritidis in the presence of CFCS of KSBT 56. Based on these results, we proposed that the CFCS of the KSBT 56 strain might secrete components which can down regulate virulence related genes in *S.* Enteritidis. The reduced expression of genes involved in invasion is therefore one of the important mechanisms which contributes to the antimicrobial effect of probiotics on intestinal pathogens. Overall, the results obtained from this study indicate that, the KSBT 56 strain isolated from fermented milk product can serve as a putative probiotic with effective anti-microbial properties. The *in vitro* data suggests that the isolated KSBT 56 strain might exert its beneficial effect via multifactorial mechanisms, which might act synergistically to antagonize intestinal pathogens. In the present study, we have provided key insights into possible mechanism of action of the KSBT 56 strain against *S.* Enteritidis and established its beneficial properties as a probiotic strain, which can be further exploited for commercial purposes.

## Conclusions

Overall results from this study suggested that KSBT 56 strain showed a potent antimicrobial activity against *S.* Enteritidis. The KSBT 56 strain was found to considerably inhibit the growth, adherence and invasion of *S*. Enteritidis. Similarly, the biofilm forming ability of *S.* Enteritidis was substantially reduced by the KSBT 56 strain. The structures of the nonbacteriocin and non-lactic acid components and the specificity of their antagonistic activity against enteroinvasive and enterovirulent *S.* Enteritidis strain remain an important area of future research.

## Methods

### Bacterial strains and culture conditions

KSBT 56 strain was isolated from *dahi chenna* (traditional fermented milk product) obtained from a local household. *L. plantarum* MTCC 1407 was used as a reference strain. *Lactobacillus* strains were grown in deMan, Rogosa and Sharpe (MRS) (HiMedia Pvt. Ltd., Mumbai) broth under aerobic conditions at 37°C for 18 h. *S.* Enteritidis was grown for 12 h and subcultured in Luria-Bertani LB (HiMedia Pvt. Ltd., Mumbai) at 37°C and used until they reached the early log phase of growth. For, biofilm, adhesion and invasion assays, equivalent cfu/ml counts of live KSBT 56 and *S.* Enteritidis cultures were used to determine the competitive exclusion of the pathogen and a sub-lethal dose of CFCS was used to determine the effect of CFCS on adhesion and invasion of the pathogen. Preliminary experiments confirmed M-17 medium to be an appropriate medium for co-culture experiments with *S.* Enteritidis and the live KSBT 56 strain. The bacterial strains used in this study are listed in Table 2.

**Table 2:** Bacterial strains used in the study

**Strain name**	**Culture collection number**	**Reference or source**
*S.* Enteritidis P125109	ATCC 13076	[36]
*Lactobacillus plantarum*	MTCC 1407	A kind gift from Dr. Knut Heller
KSBT 56	NCDC 681	Isolated from dahi chenna, a traditional food product of India
*Lactobacillus fermenti*	ATCC 9338	ATCC
*Lactobacillus brevis*	ATCC 367	ATCC
*Lactobacillus casei*	ATCC 9595	A kind gift from Dr. Peter Leuthy

### Preparation of cell free culture supernatant

CFCS of the probiotic strains are generally preferred over live probiotic bacteria for *in vitro* inhibition assays because probiotics have longer lag phase and generation time than *S*. Enteritidis. Further, *Salmonella* growth would be favoured before the probiotic strain could express its antimicrobial activity. Therefore, the CFCS of KSBT 56 strain was taken for the inhibition assays against *S*. Enteritidis. The CFCS of KSBT 56 strain was prepared as described by Truusalu *et al.*[6]. Briefly, cells were grown overnight in MRS broth for 18 h. KSBT 56 culture was centrifuged at 15000 rpm for 20 min and CFCS was filter-sterilized using 0.22-μm-pore-size millipore filters (Millipore Co., Italy).

### Cell cultures

HCT-116 colon cells were grown in Dulbecco’s modified Eagle Medium (DMEM) (HiMedia Pvt. Ltd., Mumbai) supplemented with 10% inactivated fetal bovine serum (FBS), glutamine (1.5 mM/500 ml) and penicillin (0.2 U/ml), streptomycin (0.1mg/ml). Cells were cultured at 37°C in an atmosphere of 5% CO_2_ and 95% air.

### Effect of CFCS on viability of *Salmonella*

*S.* Enteritidis culture transformed with pCJLA plasmid expressing green fluorescent protein (GFP) was grown overnight and subcultured for 2 h. CFCS of the KSBT 56 strain was added in increasing concentration to the *S.* Enteritidis culture in an early exponential phase and incubated further for 3 h. The bacterial cells were pelleted by centrifugation (1500 rpm for 5 mins) washed and resuspended in phosphate-buffered saline (PBS) and stained with propidium iodide. Flow cytometric analysis of the dead and live *S.* Enteritidis was carried out to analyze the inhibitory activity of the CFCS of the KSBT 56 strain. Flow cytometric measurements were performed using a FACScanto™ II cytometer (Becton–Dickinson, Erembodegem, Belgium). First, unstained *S*. Enteritidis WT strains were used to set the photo multiplier tube (PMT) voltage of flow cytometer and distinguish bacteria from debris. Subsequently, *S.* Enteritidis expressing GFP and those stained with propidium iodide were detected on separate channels after setting the compensation control. The results were analyzed using Flowjo software (Vx 10.0.6 beta).

### Effect of CFCS of the isolated KSBT 56 strain on other *Lactobacillus* strains

To determine the effect of the CFCS on other probiotic strains, overnight culture of *Lactobacillus casei, Lactobacillus fermenti* and *Lactobacillus brevis* were co-cultured with the CFCS of the probiotic strain at 37°C at minimum inhibitory concentration (11% CFCS of KSBT 56) determined for *S.* Enteritidis earlier. The analysis of growth was based on OD measurements at 600 nm determined at baseline and after 24 h of incubation. Each experiment was performed in triplicates and repeated thrice.

### Determination of lactic acid concentration

Lactic acid is the known component secreted by probiotic strains involved in the inhibition of enterocolitic pathogens. To determine whether the isolated KSBT 56 strain was producing lactic acid equivalent to other reference strains like *L. plantarum* MTCC 1407, a commercially available D- and L- Lactic acid estimation kit (Megazyme, Ireland) was used. After culturing the KSBT 56 and the reference strain for 6 h at 37°C, lactic acid concentration was determined by D- and L- Lactic acid estimation kit according to manufacturers instructions. The lactic acid concentration in the CFCS of KSBT 56 strain was also estimated in a similar manner, to determine if the inhibitory activity of CFCS was due to the production of lactic acid.

### Determination of the antimicrobial activity of free radicals of the KSBT 56 strain

To determine the antimicrobial activity of the free radicals produced by KSBT 56 strain against *S.* Enteritidis, superoxide dismutase gene (*sodC*) knock out mutant was used*. sodC* gene product is known to neutralize the effect of free radicals and protect the bacteria. One step inactivation method was used to construct a knock out mutant of *S.* Enteritidis WT by deleting the *sodC* gene [34]. Briefly, PCR primers providing homology to *sodC* gene were used to knock out the gene. An easily curable, low copy number plasmid pKD46 was used to facilitate homologous recombination of the PCR primers with homology to the *sodC* gene and template plasmid (pKD4) carrying kanamycin resistance genes was transformed into *S.* Enteritidis. The primers used in the study are listed in Table 3. The mid log phase growth of *S.* Enteritidis WT strain and *sodC* gene knockout mutant was subcultured with 7% CFCS of the KSBT 56 strain, for 4 h. It was determined in earlier experiment that 7% CFCS of KSBT 56 strain considerably inhibited the growth of *S.* Enteritidis. Similarly, both the strains were co-cultured with the live KSBT 56 strain in M-17 medium. The cfu counts were enumerated by plating appropriate dilutions of the above groups in LB agar plates supplemented with streptomycin (50 μg/ml).

**Table 3:** Primers used in the study

**Primer**	**Sequence (5′ to 3′)**	**Reference**
*hilA* F	TTAACATGTCGCCAAACAGC	[37]
*hilA* R	GCAAACTCCCGACGATGTAT	[37]
16s rRNA F	GATCATGGCTCAGATTGAACGCTGGCGG	[37]
16s rRNA R	CACCGCTACACCTGGAATTATACCCCCTC	[37]
FwKOSenSodC	TTTTATGGGTAAAACGAAATTATGACGATATGGCTATGTTGCTGTGTGTAGGCTGGAGCTGCTTC	In this study
RwKOSenSodC	TTTTATTAATGGTATTTACGATACAACCAAAAAACGAGGTAACTAATATGAATATCCTCCTTAGTT	In this study

### Effect of KSBT 56 strain on biofilm formation

The biofilm formation by *S.* Enteritidis was assessed by incubating *Salmonella* with the probiotic strain in a 96 well plate for 24 h. The experiment was performed in the following groups: **Group A:***S.* Enteritidis (10^8^ cells/ml) **Group B:***S.* Enteritidis + KSBT 56 strain in the ratio of 1:1. **Group C:***S.* Enteritidis was added 1 h after the addition of the KSBT 56 strain in the ratio of 1:1. The biofilm formation by *S*. Enteritidis in the above wells was confirmed by crystal violet staining. The wells were washed with PBS thrice. Subsequently, the biofilm forming ability of *Salmonella* in various groups was determined by plating and enumeration of adherent bacteria in 96 well plates on LB Agar supplemented with streptomycin (50 μg/ml). The bacteria adhered to the wells forming biofilms were scrapped and different dilutions were plated. The plates were incubated at 37°C for 24 h and cfu count recovered from the biofilms was determined. KSBT 56 strain was included as a control in the experiment.

### Invasion assay

Invasion of *S.* Enteritidis to HCT-116 cell line was carried out as previously described [35], with minor modifications. Briefly, HCT-116 cell line was maintained in DMEM and passaged until confluence. The monolayer cells were seeded on 24 well tissue culture plates (Nest Biotech, China) and the confluent cells were washed thrice with PBS. *S.* Enteritidis was grown overnight and subcultured for 4 h in LB medium [36]. Bacterial cells were washed and resuspended in DMEM and infected to HCT-116 cell lines at a multiplicity of infection (MOI) of 100:1. The experiment was performed on 24 well plates in various groups. **Group A:***S.* Enteritidis (1 × 10^8^ cells/ml) **Group B:***S.* Enteritidis + KSBT 56 in the ratio of 1:1. **Group C:***S.* Enteritidis was added 1h after the addition of the KSBT 56 strain in the ratio of 1:1. **Group D:***S.* Enteritidis + *L. plantarum* MTCC 1407 (1:1), was taken as control. The plate was incubated for 50 min at 37°C in CO_2_ incubator. HCT-116 cells were further incubated for 2 h in media containing gentamicin (100 μg/ml). Infected cells were washed twice with PBS and lysed with 0.1% Triton X-100. Dilutions of the resulting cell lysates were plated on streptomycin LB Agar for determination of intracellular bacterial counts. The above groups were also processed for confocal microscopy for supportive evidence of invasion assay**.** In a separate experiment, to determine the effect of CFCS on *Salmonella* invasion, *S.* Enteritidis was either co-incubated with CFCS (sub-lethal dose of 5% of CFCS) or added after culturing with CFCS for 1 h, to a 24-well tissue culture plate seeded with HCT-116 cells and standard gentamicin protection assay was performed as described above.

### Confocal microscopy

HCT-116 monolayers were incubated overnight at 37°C in a humidified atmosphere at 5% CO_2_ in cell culture medium without antibiotics before the addition of bacteria (MOI, 50:1). After incubation for 50 min in an appropriate medium without fetal bovine serum, cells were washed in PBS to remove non-invading bacteria. The monolayer cells, prepared on glass coverslips, in 24 well tissue culture plates (Nest Biotech, China), were fixed with 4% paraformaldehyde (PFA) and then stained with plasma red dye (Invitrogen, Green Island, USA). DAPI was used to stain the nucleus of HCT-116 cells. *S.* Enteritidis containing plasmid pCJLA expressing GFP was visualized using Confocal Laser Scanning Microscope (CLSM, Leica). Z-stacking was used to distinguish the internalized bacteria from the extracellular bacteria.

### Adhesion assay

Adhesion assay was carried out as described previously [14]. Each well of a 24-well tissue culture plate was seeded with HCT-116 cells. 500 μl of DMEM without serum and antibiotics was added to each well and incubated at 37°C for 30 min. *S.* Enteritidis was grown overnight and the experiment was performed in the following groups. **Group A:***S.* Enteritidis, 1 × 10^8^ cfu/ml **Group B:** KSBT 56, 1 × 10^8^ cfu/ml **Group C:***S.* Enteritidis: KSBT 56 (1:1) **Group D:***S.* Enteritidis added 1 h after the addition of KSBT 56**.** Plate was incubated for 20 min at 4°C and the cells were detached by adding Trypsin EDTA solution (HiMedia Pvt. Ltd., Mumbai). The cells were further incubated for 15 min at room temperature. The cell suspensions from each group were plated at appropriate dilutions on MRS agar and LB Agar supplemented with streptomycin for differential growth of KSBT 56 and *S.* Enteritidis. Similarly, the effect of CFCS on adhesion was determined by co-incubating *S.* Enteritidis with CFCS in 24-well tissue culture plate seeded with HCT-116 cells or adding *S*. Enteritidis to the wells after 1 h of subculturing with CFCS, and adopting the above protocol of adhesion assay. A sub lethal dose of 5% CFCS of KSBT 56 was used for the assay.

### Expression analysis of hilA gene (SPI1) by RT-PCR

Probiotics are known to down-regulate the expression of virulence genes of *S.* Enteritidis present in both SPI1 and SPI2. *hilA* gene is the major transcriptional regulator of SPI1 and down-regulation of *hilA* reflects the down-regulation of SPI1 genes required by *S.* Enteritidis for successful invasion into host epithelial cells [37]. To study the SPI1 regulation by KSBT 56, *S.* Enteritidis culture was grown overnight and subcultured for 4 h in the presence of increasing concentration of CFCS of the KSBT 56. RNA was isolated using Real Genomics RNA mini kit (Real Biotech Corporation, India) as per the manufacturers instructions and reverse transcribed to cDNA using cDNA synthesis kit (Fermentas, USA). The relative quantification of *hilA* gene expression was analyzed by using 16s rRNA as the reference gene for both treated and untreated *S.* Enteritidis culture. The RT-PCR was carried out by using SYBR Green Master Mix (Roche Applied Science, Mumbai, India). The PCR reaction conditions consisted of initial denaturation at 95°C for 5 min, 40 cycles of denaturation at 95°C for 15 sec, followed by annealing at 54°C for 30 sec and extension at 72°C for 45 sec. The primers used in the experiment are listed in Table 3.

### Statistical analysis

All the data represent the mean ± standard deviation of three independent experiments*.* The significant differences in the various experimental groups were determined by *t*-test with the help of GraphPad Prism software version 5. The flow cytometric data analysis was carried out by using Flowjo V× 10.0.6 beta.

## Abbreviations

S. Enteritidis: *Salmonella* enterica serovar Enteritidis; SEn: *Salmonella* Enteritidis PBS, Phosphate-Buffered Saline; SPI: Salmonella Pathogenicity Island; CFCS: Cell Free Culture Supernatant; CLSM: Confocal Laser Scanning Microscopy; L. plantarum: *Lactobacillus plantarum*; WT: Wildtype; GFP: Green Fluorescent Protein.

## Competing interests

The authors declare that they have no competing interests.

## Authors contributions

MS and JK conceived and designed the experiments. JK, DM and PR performed the experiments. TKB performed the confocal microscopy experiments. MS, NS and PT coordinated the study. NS and JK drafted the manuscript. All authors finally read and approved the manuscript.
